# Advanced liver-on-chip model mimicking hepatic lobule with continuous microvascular network via high-definition laser patterning

**DOI:** 10.1016/j.mtbio.2025.101643

**Published:** 2025-03-07

**Authors:** Masafumi Watanabe, Alice Salvadori, Marica Markovic, Ryo Sudo, Aleksandr Ovsianikov

**Affiliations:** a Research Group 3D Printing and Biofabrication, Institute of Materials Science and Technology, Technische Universität Wien (TU Wien), 1060 Vienna, Austria; b Austrian Cluster for Tissue Regeneration (https://www.tissue-regeneration.at), Austria; c Japan Society for the Promotion of Science (JSPS) Overseas Research Fellow, Japan; d Department of System Design Engineering, Keio University, 223-8522 Yokohama, Japan

**Keywords:** Liver-on-chip, Microphysiological systems, Vascularization, Hepatic lobule, Laser patterning, Toxicology, High Definition (HD)

## Abstract

There is a great demand for development of advanced *in vitro* liver models to predict the efficacy and safety of drug candidates accurately in the preclinical drug development. Despite the great efforts to develop biomimetic models, it remains challenging to precisely mimic a functional unit of the liver (i.e., hepatic lobule) with a continuous microvascular network. Recent progress in laser patterning has allowed us to create arbitrary biomimetic structures with high resolution. Here, we propose an advanced liver-on-chip model mimicking the hepatic lobule with a continuous microvascular network, ranging from the microvessels to the central vein of the liver, utilizing femtosecond laser patterning. Firstly, we optimize the laser power to pattern microchannels mimicking the microvessel and central vein of the hepatic lobule by using a femtosecond laser within a collagen-based hydrogel containing hepatic cells. Secondly, we construct continuous microvessels with luminal structures by comparing different microchannel sizes in diameter. Finally, we assemble a millimeter-scale hepatic lobule-like structure with multiple layers of microvascular networks in the liver-on-chip. Furthermore, our liver-on-chip model exhibits major liver functions and drug-induced hepatotoxicity, as evidenced by albumin and urea productions and by a toxic response to acetaminophen, respectively. Our approach provides valuable strategies for the development of advanced physiological and pathological liver-on-chip models for pharmaceutical and toxicological studies.

## Graphical abstract

**Figure:**
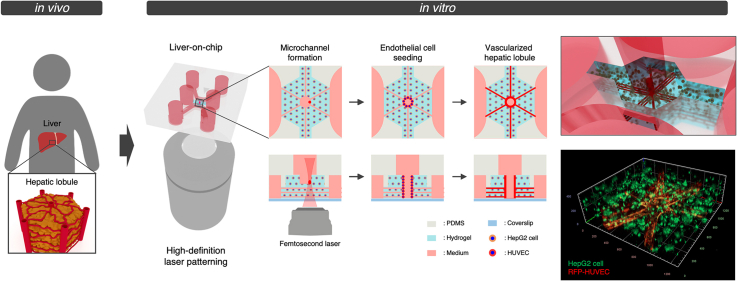

## 1 Introduction

The current drug development process largely relies on animal models to assess the efficacy and safety of drug candidates in the preclinical stage [1]. However, data from animal models frequently fails to predict the results obtained in human clinical stage, including life-threatening toxicity and ineffective performance of drug candidates, primarily due to interspecies differences [2,3]. In particular, unexpected drug-induced liver injury in humans is one of the major obstacles in the drug development process and a leading cause of drug withdrawal from the market [4]. Therefore, there is a growing demand for development of *in vitro* humanized liver models that can replicate the liver microstructures and functions in order to better predict the efficacy and safety of drug candidates.

To construct functional liver tissue *in vitro*, researchers have focused on a minimum functional unit of the *in vivo* liver, known as the “hepatic lobule” [5]. The hepatic lobule is mainly composed of a high hepatocyte-density tissue and a continuous vascular network with dimensions ranging from microvessels to a large vessel (e.g., central vein). Over the last decades, various three-dimensional (3D) biofabrication techniques have been utilized, such as cell spheroid [[6], [7], [8]], organoid [9,10], microfluidic [[11], [12], [13]], and bioprinting techniques [14,15] to mimic the complexity of the hepatic lobule. For example, a microfluidic technique enabled the construction of a 3D hexagonal hepatic lobule with 2D endothelial cell (EC) networks in a multi-layered chip [16]. On the other hand, stereolithography-based bioprinting realized 3D hydrogel-based models that patterned human hepatocyte-like cells and ECs in a hepatic lobule-like structure through photopolymerization of the hydrogel [17,18]. Moreover, a recent work utilizing extrusion-based bioprinting demonstrated 3D hepatic lobule-like structures with a large vessel mimicking the central vein [19]. Despite these great efforts, it remains challenging to construct a continuous hierarchical vascular network with luminal structures in a hepatic lobule-like structure, especially smaller than 100 μm in diameter, by utilizing conventional techniques. Given that drug candidates are delivered from portal veins to hepatocytes through adjacent microvessels in the liver, there is a critical need for the construction of continuous vascular network ranging from large vessels to microvessels in order to obtain biomimetic 3D liver models.

Recent progress in high-definition (HD) bioprinting enabled realization of complex 3D structures reproducibly and high resolution utilizing laser patterning [20]. The use of femtosecond lasers allows to achieve the finest resolution (<1 μm) that allows crosslinking or other structural modifications within transparent cell-containing materials. Recent trends in this field have addressed biomedical applications to realize the *in vivo* complexity of organ/tissue microstructures, such as microvessels, using synthetic [[21], [22], [23]] and natural hydrogels [[24], [25], [26], [27]]. In particular, these recent works utilizing femtosecond laser photoablation have demonstrated promising results to construct arbitrary 3D microvascular structures in natural hydrogels with high resolution and reproducibility. More recently, we have established a protocol to construct physiologically relevant microvascular structures in a collagen-based natural hydrogel in a microfluidic chip by utilizing HD laser patterning [28]. Notably, the constructed microvessels showed perfusable and continuous luminal structures and physiological barrier function comparable to the *in vivo* microvessels.

Here, we propose an advanced liver-on-chip model mimicking the hepatic lobule with a continuous microvascular network, from the microvessels to the central vein of the liver utilizing HD laser patterning. To realize our conceptual design of liver-on-chip, we first create microchannels mimicking the microvessels and the central vein of the hepatic lobule by patterning hepatic cell-containing hydrogels directly within a pre-assembled microfluidic chip. In particular, in order to pattern the controlled microchannels in the high cell-density hydrogel, we introduce a photosensitizer to boost the efficiency of laser patterning. Moreover, we optimized not only the concentration of the photosensitizer, but also the laser power to achieve microchannel formation corresponding to the initial 3D CAD model. After the optimization of laser settings, ECs are introduced to the laser-patterned microchannels to construct a continuous microvascular network. To address this, microchannels of different size are compared. Finally, we produced a millimeter-scale hepatic lobule-like structure with the continuous microvascular network, including the microvessels and the central vein, in the liver-on-chip. Furthermore, levels of major liver functions were measured in order to assess our proposed liver-on-chip model. Interestingly, albumin and urea productions were upregulated in the presence of laser-patterned microchannels. Additionally, we confirmed cellular damage in response to a drug, acetaminophen (APAP), indicating that our liver-on-chip model has the ability to recreate an acute liver failure process. Taken together, our results support that our proposed liver-on-chip model will be a promising tool for preclinical pharmaceutical and toxicological studies.

## 2 Materials and methods

Unless stated otherwise, all the chemicals were purchased from Sigma-Aldrich (Saint Luis, MO, USA).

### 2.1 Cell culture

HepG2 cells were obtained from American type culture collection (ATCC; Manassas, VA, USA) and cultured in HepG2 medium, which was composed of minimum essential medium Eagle supplemented with 10 % fetal bovine serum (FBS; Gibco, Carlsbad, CA, USA) and 1 % penicillin-streptomycin (Merck, Darmstadt, Germany). The cells were expanded in culture flasks in a humidified 5 % CO_2_ incubator at 37 °C and the culture medium was replaced every 3–4 days. Human umbilical vein endothelial cells (HUVECs) were obtained from Lonza (Basel, Switzerland) and red fluorescent protein-expressing HUVECs (RFP-HUVECs) were obtained from PeloBiotech (Planegg, Germany). They were cultured in endothelial growth medium-2 (EGM-2; Lonza) supplemented with additional 3 % FBS in order that the final concentration of FBS was adjusted to 5 %. The cells were expanded in culture flasks coated with quick coating solution (PeloBiotech) in a humidified 5 % CO_2_ incubator. The culture medium was replaced every 2–3 days.

### 2.2 Microfluidic chip

A master mold of microfluidic chip was designed in AutoCAD (Autodesk, San Francisco, CA, USA) in order to realize the millimeter-scale hepatic lobule in the central channel of the chip. To create the master mold, an aluminum block was processed using an automated 5-axis milling machine (D200Z, Makino, Tokyo, Japan), resulting in a negative master mold of the chip. The detailed fabrication process of the microfluidic chip by soft lithography was previously described [29,30]. Briefly, polydimethylsiloxane (PDMS; Sylgard 184, Dow Corning, Midland, MI, USA) was casted on the master mold and then cured in an oven to copy the chip design. The cured PDMS replica was removed from the mold, trimmed, punched to form inlets/outlets, and then autoclaved. The autoclaved PDMS replica was bonded to a coverslip (thickness #1, Carl Roth, Karlsruhe, Germany) to form microfluidic channels for hydrogel and culture medium. The PDMS microfluidic chip was sterilized under UV light before use. To avoid the detachment of the hydrogel from the PDMS channel, the microfluidic chip was modified with 3-aminopropyltriethoxysilane (APTES)-polydopamine (PDA) hybrid, rendering the channel hydrophilic [31,32]. The chip was incubated with 1 %(v/v) of APTES for 20 min to increase the strength of adhesion between the PDMS channel and PDA, followed by rinsed with 70 % isopropanol and water once each. Subsequently, the chip was incubated with 2 mg/mL of dopamine solution prepared in 10 mM Tris-HCl (pH 8.5) for 2 h to increase the strength of adhesion between the PDMS channel and the hydrogel [33]. Finally, the microfluidic chip was rinsed with sterile water three times and dried in an oven.

### 2.3 Cell metabolic activity assay in 2D with photosensitizer

Cell metabolic activity was measured using PrestoBlue assay (Invitrogen, Carlsbad, CA, USA). HepG2 cells were seeded in a 96-well plate and allowed to attach to the well plate for 24 h. A photosensitizer, tetrapotassium 4,4-(1,2-ethenediyl)bis[2-(3-sulfophenyl)diazenesulfonate] (DAS) [34] dissolved in HepG2 medium was added to the well plate at different concentrations up to 4 mM and then the cells were incubated for 24 h. Thereafter, PrestoBlue reagent was diluted 1:10 with HepG2 medium and incubated with the cells for 1 h. The absorbance was measured with a plate reader (BioTek Synergy H1, Agilent, Santa Clara, CA, USA) at an excitation wavelength of 560 nm and an emission wavelength at 590 nm.

### 2.4 3D encapsulation of HepG2 cells in hydrogel in microfluidic chip

A rat-tail type I collagen (Corning, New York, NW, USA) was adjusted to a concentration of 4 mg/mL at pH 7.0. The neutralized rat-tail collagen and Matrigel (Growth factor reduced, Corning) were mixed at 3:1 ratio. Thereafter, HepG2 cells were detached from culture flasks using 0.25 % Trypsin-EDTA and then encapsulated in the collagen-Matrigel pre-gel solution at a density of 0.5 × 10^6^ cells/mL. A 10-μl cell suspension of HepG2 cells in the hydrogel was filled in the central channel of the microfluidic chip (on day 0). The chip samples were placed in a humidified 5 % CO_2_ incubator at 26 °C for 2 h, followed by 37 °C for 1 h for gelation, as previously described [28]. After gelation of the hydrogel, the side channels and side reservoirs were filled with HepG2 medium. The cells were cultured under the static condition where delivery of culture medium relies solely on diffusion from the side channels and the side reservoirs in all experiments. The culture medium was replaced every day. In order to perform live-cell imaging of HepG2 cells in the hydrogel, the cells were fluorescently labeled with 5 μM of BioTracker 488 Green CFSE cell proliferation kit at 37 °C for 15 min. Subsequently, the cells were encapsulated in the Collagen-Matrigel pre-gel solution and filled in the central channel of the microfluidic chip as described above.

### 2.5 Femtosecond laser patterning in hydrogel containing HepG2 cells

Laser patterning was performed using a femtosecond near-infrared (NIR) laser (MaiTai eHP DeepSee, Spectra-Physics, Milpitas, CA, USA) with a pulse duration of 70 fs after the microscope 10 × /0.4 objective lens (UPlanSApo, Olympus, Tokyo, Japan) and a repetition rate of 80 MHz. A microscope stage (ScanPLUS IM 120 × 180, Märzhäuser Wetzlar, Wetzlar, Germany) was implemented to position the sample and a dual-axis galvanometric scanner (Scanlabs, Puchheim, Germany) was used to position the laser beam within the field of view [22,35]. 3D data for microchannels were created in AutoCAD and exported as STL files. The system was controlled by Think3D software from UpNano GmbH (https://www.upnano.at/software). Microchannels mimicking the vasculatures were directly patterned within the hydrogel containing HepG2 cells in the microfluidic chip by the femtosecond laser. All experiments were performed with constant writing speed (=600 mm/s) and line spacing (=0.5 μm). The laser power was optimized in a range from 200 to 700 mW to create perfusable microchannels. As for the addition of DAS, on the following day of the cell encapsulation in hydrogel, the culture medium in the microfluidic chip was replaced with a fresh medium containing 0.5 mM DAS for longer than 2 h before the laser patterning (on day 1). After the laser patterning, the medium containing DAS was replaced with a fresh medium without DAS.

### 2.6 Visualization of laser-patterned microchannels

Fluorescein isothiocyanate-labeled dextran (FITC-Dextran, 2000 kDa) dissolved in PBS at 1.0 mg/mL was added to the laser-patterned microchannels in a microfluidic chip on the following day of the laser patterning. 3D fluorescence images of FITC-Dextran were obtained using a laser-scanning confocal microscope (LSM800, Carl Zeiss, Oberkochen, Germany). The width, height, and area of the laser-patterned microchannels were measured in ImageJ (National Institutes of Health, Bethesda, MD, USA).

### 2.7 Cell viability assay in 3D hydrogel after laser patterning

The viability of HepG2 cells was evaluated by staining with Hoechst 33342 (Invitrogen, Waltham, MA, USA) and propidium iodide (PI) for all cell nuclei and damaged cell nuclei, respectively. Hoechst and PI were diluted 1:1000 and 1:1250 with PBS. The chip samples were incubated with the diluted Hoechst and PI solution for 60 min at 37 °C. Fluorescence images were obtained using the laser-scanning confocal microscope (LSM800).

### 2.8 Vascularization of liver-on-chip

The seeding process of RFP-HUVEC in the present study has been previously described [28]. Briefly, after RFP-HUVECs were detached from culture flasks using 0.05 % Trypsin-EDTA, the cells were suspended in EGM-2 (5 % FBS) to adjust to a cell density of 5 × 10^6^ cells/mL. A 10-μl cell suspension of RFP-HUVECs was seeded on the microfluidic chip. The microfluidic chip was tilted and then placed in a humidified 5 % CO_2_ incubator at 37 °C for 30 min to allow the cells to attach to the surface of the Collagen-Matrigel hydrogel. Thereafter, the side channels and side reservoirs of the microfluidic chip were filled with EGM-2 (5 % FBS). The cells were cultured under the static condition and the culture medium was replaced every day. 3D fluorescence images of microvessels and bright-field images of the hydrogel region were obtained using the laser-scanning confocal microscope (LSM800). The length and width of the microvessels in fluorescence images and the area of HepG2 cell aggregates in bright-field images were measured in ImageJ.

### 2.9 Albumin and urea assays

To assess liver function of the chip samples, supernatants of culture medium in microfluidic chips were collected on days 5 and 9 and stored at −20 °C until measurement. Albumin production was measured using a human albumin enzyme-linked immunosorbent assay (ELISA) kit (Invitrogen), while urea production was measured using a urea assay kit (abcam, Cambridge, UK) according to the manufacturer's instructions.

### 2.10 Drug-induced hepatotoxicity test

Hepatotoxicity test was performed using acetaminophen (N-acetyl-para-aminophenol, APAP) as a model drug. APAP was administered to the liver-on-chip at a concentration of 4 mM on day 9 for 24 h (=APAP(+) group). As APAP was dissolved in ethanol, the same amount of ethanol as the APAP(+) group was added to culture medium in chip samples in the control group (= APAP (−)). After the APAP treatment, PrestoBlue assay and live/dead staining were performed to evaluate cell metabolic activity and cell viability. PrestoBlue reagent diluted 1:10 with EGM-2 was added to the chip samples and incubated for 1 h. The reacted PrestoBlue reagent was collected from the chip samples and its absorbance was measured with the plate reader (BioTek Synergy H1) as described in the “cell metabolic activity assay in 2D”. After that, the chip samples were stained with Hoechst and PI as described in the “cell viability assay in 3D”. The numbers of cells positive for Hoechst and negative for PI were manually counted in ImageJ to calculate the percentage of live cells in the liver-on-chip.

### 2.11 Statistical analyses

Statistical analyses were performed using the statistical software R (version 4.2.3, R Core Team, Vienna, Austria). Data is presented as mean ± standard deviation (SD). We assumed a normal distribution of data. Student's *t*-test was used to compare the differences between two groups. One-way or two-way analysis of variance (ANOVA), followed by Dunnett's test or Tukey post hoc test, was used to compare the differences between more than three groups. *P*-value of <0.05 was considered statistically significant.

## 3 Results

### 3.1 Design of liver-on-chip

In order to construct a minimum functional unit of the *in vivo* liver (i.e., hepatic lobule) (Fig. 1A), the microfluidic chip was designed to possess a millimeter-scale central channel to mimic the hexagonal hepatic lobule and two side channels to supply culture medium to the cells in the central channel (Fig. 1B–S1). Since the *in vivo* hepatic lobule is composed of a high hepatocyte-density tissue and a microvascular network, hepatic cells (i.e., HepG2 cells) were encapsulated in a hydrogel to construct the hepatocyte tissue, while HUVECs were seeded to laser-patterned microchannels to construct a vascularized hepatic lobule-like structure in a microfluidic chip.

**Fig. 1:**
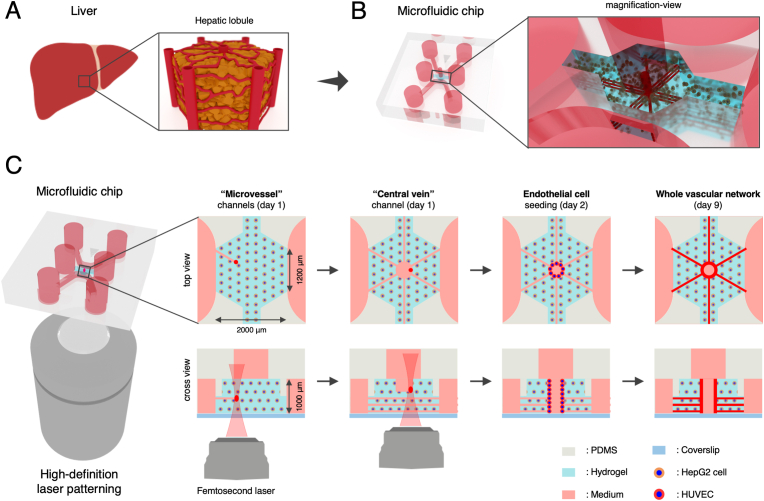
Concept design of liver-on-chip via femtosecond laser patterning. (A) Schematic illustrations of minimum functional unit of the *in vivo* liver (i.e., hepatic lobule), which is composed of a dense-hepatocyte tissue (brown) and a microvascular network (red). (B) Construction of the hepatic lobule-like structure in a microfluidic chip. (C) Fabrication process of the liver-on-chip via high-definition (HD) laser patterning. (For interpretation of the references to colour in this figure legend, the reader is referred to the Web version of this article.)

To realize our design of liver-on-chip, the central channel (width = 2000 μm and height = 1000 μm) of the microfluidic chip was filled with a collagen-based hydrogel containing HepG2 cells, while two side channels were filled with culture medium (day 0). Microchannels mimicking the hepatic microvascular networks, including the microvessels and the central vein, were then patterned within the dense HepG2 cell tissue by the femtosecond laser (day 1). Subsequently, HUVECs were seeded to the laser-patterned microchannels for vascularization of the liver-on-chip (day 2). Finally, a vascularized hepatic lobule-like structure was constructed in the hydrogel region (day 9) (Fig. 1C).

### 3.2 Formation of “microvessel” and “central vein” channels in hydrogel containing HepG2 cells

To create the laser-patterned microchannels within the cell-containing hydrogel in a more efficient manner, an effect of a biocompatible diazosulphonate photosensitizer (i.e., DAS [34]) on microchannel formation was investigated. For evaluation of appropriate DAS concentration, the cytocompatibility of DAS with HepG2 cells was assessed in 2D. After 24 h of incubation with DAS, the HepG2 cells exhibited normal metabolic activity with lower concentrations of DAS (≤3 mM), while 4 mM of DAS resulted in a significant decrease in metabolic activity (Fig. S2). For this reason, DAS concentrations at 0.5, 1, and 2 mM were used in the following 3D chip experiment. After the laser patterning, the “microvessel” channels were visualized by FITC-Dextran (2000 kDa) perfusion. Fluorescence images revealed that the addition of DAS at 0.5 mM led to clear formation of three layers of microchannels in the cell-containing hydrogel, while the control group (without DAS) required high laser powers for the microchannel formation (Fig. S3). Besides, the addition of DAS at 1 and 2 mM resulted in the diffusion of FITC-Dextran throughout the hydrogel region without showing clear microchannels (Fig. S3).

Based on the result, further analyses were carried out with 0.5 mM DAS to identify an optimal laser power for the formation of three layers of the microchannels (Fig. 2A). As predicted, the control group (without DAS) failed to create Ø50 μm channels in the cell-containing hydrogel reproducibly, especially in the 2nd and 3rd layers within the tested HD laser patterning parameter range (Fig. 2B). Quantitative analysis also revealed that the widths of the laser-patterned microchannels were much smaller than the initial CAD model. For example, even with the maximum laser power (i.e., 700 mW), the microchannel widths were 35.8 ± 22.2 μm (72 % of the initial CAD model) in the 1st layer, 31.8 ± 23.2 μm (64 %) in the 2nd layer, and 24.0 ± 24.1 μm (48 %) in the 3rd layer in the control group. By contrast, in the group with 0.5 mM DAS, the microchannels were found to be open and perfusable when patterned with less laser power compared to the control group (Fig. 2C). More importantly, the widths of the laser-patterned microchannels corresponded to the initial CAD model when the laser powers were above 300 mW in the 1st and 2nd layers, and above 400 mW in the 3rd layer (Fig. 2C). Moreover, quantitative analyses show the microchannel widths were 45.6 ± 10.1 μm (91 %) in the 1st layer, 48.5 ± 4.7 μm (97 %) in the 2nd layer, and 50.8 ± 6.0 μm (102 %) in the 3rd layer with the laser power of 500 mW. In addition, we observed slightly larger microchannel heights in the z plane than the initial CAD model. The microchannel heights were 58.6 ± 9.0 μm (117 %) in the 1st layer, 55.7 ± 14.0 μm (111 %) in the 2nd layer, and 64.3 ± 11.3 μm (129 %) in the 3rd layer with the laser power of 500 mW (Fig. S4). Furthermore, to evaluate the potential cytotoxicity of the HD laser patterning process to HepG2 cells, live/dead staining was performed after the patterning. In our model, only a few damaged cells, which were PI-positive (PI^+^), were observed in the cell-containing hydrogel 2 h after the laser patterning at 500 mW (Fig. S5). There was no statistically significant difference in live cells (%) between Channel(−) and Channel(+) regions. Taken together, 500 mW was selected for the formation of the “microvessel” channel in the following experiments.

**Fig. 2:**
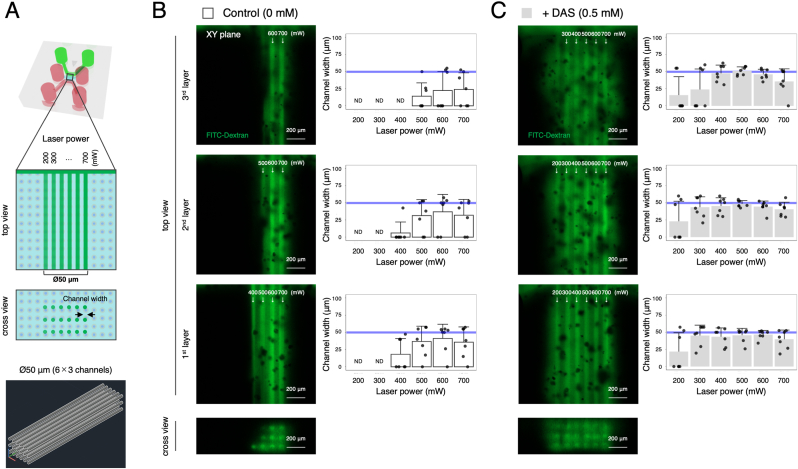
Optimization of laser power for the formation of “microvessel” channel. (A) Schematic illustrations of microchannel formation in the cell-containing hydrogel with different laser powers ranging from 200 to 700 mW and corresponding 3D CAD design. (B, C) Microchannel formation in the control group without DAS (B) and in the group with DAS at 0.5 mM (C). Fluorescence images of FITC-Dextran perfusion (2000 kDa, green) in the 1^st^, 2^nd^, and 3^rd^ layers. Scale bars: 200 μm. Quantitative analysis of channel width. Data represent the mean ± SD (n = 7/group). ND indicates “not detected”. Blue lines indicate 50 μm in channel width. (For interpretation of the references to colour in this figure legend, the reader is referred to the Web version of this article.)

Next, to create a vertical “central vein” channel, laser power was optimized in a similar manner. To this end, a 3D model of the central vein (Ø200 μm × 1000 μm) was divided into six sectors in the CAD software to compare different laser powers simultaneously in the same microfluidic chip (Fig. 3A). On the following day of the laser patterning, FITC-Dextran was added to the central inlet of the microfluidic chip and observed under the laser-scanning confocal microscope. Fluorescence images show that the microchannels were perfusable with FITC-Dextran (2000 kDa) at laser powers down to 400 mW (Fig. 3B). Moreover, quantitative analysis also revealed that channel area corresponded to the CAD model when the laser power was ≥400 mW (Fig. 3C). Mean channel areas were 114 %, 114 %, 120 %, and 131 % with the laser powers of 400, 500, 600, and 700 mW, respectively. Based on the result, 500 mW was selected to ensure the formation of the “central vein” channel.

**Fig. 3:**
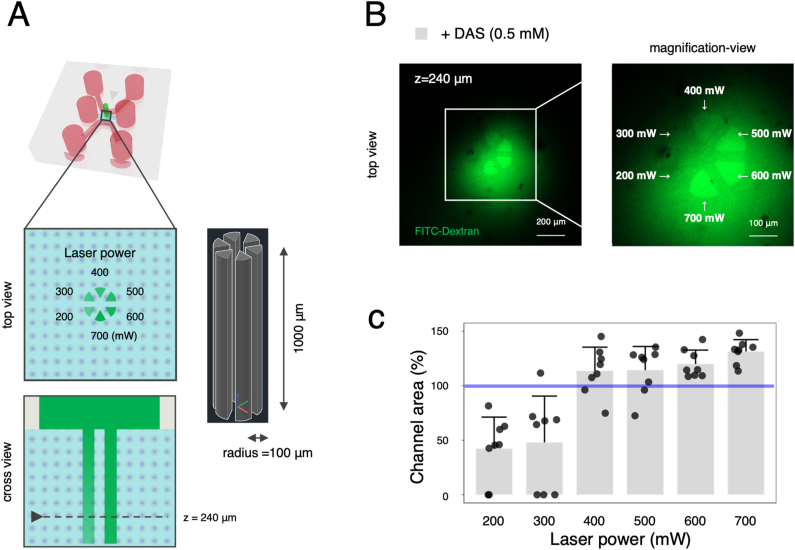
Optimization of laser power for the formation of “central vein” channel. (A) Schematic illustration of microchannel formation in the cell-containing hydrogel with different laser powers ranging from 200 to 700 mW and corresponding 3D CAD design. (B) Microchannel formation with DAS at 0.5 mM. Fluorescence images of FITC-Dextran perfusion (2000 kDa, green) at 240 μm high in z axis. Scale bars: 200 and 100 μm. (C) Quantitative analysis of channel area. Data represent the mean ± SD (n = 8/group). Blue line indicates 100 % in channel area (%). (For interpretation of the references to colour in this figure legend, the reader is referred to the Web version of this article.)

### 3.3 Realization of controlled microvascular network in liver-on-chip

To achieve vascularization of the liver-on-chip, several types of culture medium were compared to identify an optimal coculture medium for both HepG2 cells and HUVECs. In order to monitor the morphology of microvessels, RFP-HUVECs were seeded into the HD laser-patterned microchannels in the HepG2 cell-containing hydrogel, which were cultured under different conditions of culture medium, such as HepG2 medium, EGM-2, and a mixed medium of them at 1:1 ratio (Fig. S6A). RFP-HUVECs migrated into the laser-patterned microchannels in EGM-2 and the mixed medium (1:1) on day 9, while only a few cells were observed in the hydrogel region in HepG2 medium (Fig. S6B). Importantly, luminal structures of microvessels were confirmed only in the EGM-2 condition. Meanwhile, there was no statistically significant difference between different conditions of culture medium in HUVEC migration distance nor in area of HepG2 cell aggregate (Figs. S6C and S6D). To ensure successful formation of luminal structures of microvessels, EGM-2 was selected for the following coculture experiment using HepG2 cells and RFP-HUVECs.

Furthermore, to construct continuous and controlled microvessels in the HepG2 cell-containing hydrogel, RFP-HUVECs were seeded to the laser-patterned microchannels with different diameters (i.e., Ø50 and Ø80 μm) on the following day of the laser patterning (Fig. 4A). Fluorescence images show that RFP-HUVECs formed continuous and controlled microvessels with luminal structures in the Ø80 μm channels on day 5, though a few microvessels partly regressed on day 9 (Fig. 4B). In contrast, the cells exhibited discontinuous microvessels in the Ø50 μm channels on days 5 and 9. The length of continuous microvessel in the Ø80 μm channels exhibited roughly 2.5-fold increase compared to that in the Ø50 μm channels on days 5 and 9 (Fig. 4C). Moreover, the width of microvessel in the Ø80 μm channels corresponded to the initial CAD model over time, while that in the Ø50 μm channels became much narrower than the CAD model (Fig. 4D). These results indicated that the Ø80 μm channel is a suitable size for controlled vascularization to achieve continuous lumen, rapid formation, and structural maintenance of the microvessels. For this reason, the Ø80 μm channel was selected as the diameter of “microvessel” channel in the liver-on-chip.

**Fig. 4:**
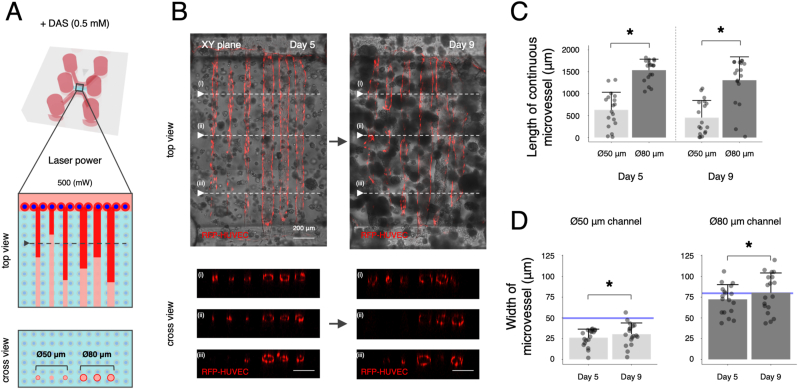
Construction of continuous microvessels in the laser-patterned microchannels. (A) Schematic illustrations of microvessel formation in the laser-patterned microchannels with different diameters (i.e., Ø50 and Ø80 μm) in the cell-containing hydrogel. RFP-HUVECs were seeded to one side channel of the microfluidic chip. (B) Fluorescence images of RFP-HUVECs (red) in the laser-patterned on days 5 and 9. The cross-view images correspond to the dotted lines (i–iii) in the above image. Scale bars: 200 μm. (C) Quantitative analysis of length of continuous microvessel on days 5 and 9. Data represent the mean ± SD (n = 18/group). ∗*p* < 0.05 (two-way ANOVA with the post hoc Tukey's honestly significant difference test). (D) Quantitative analysis of width of microvessel on days 5 and 9. Data represent the mean ± SD (n = 18/group). ∗*p* < 0.05 (Student's *t*-test). Blue lines indicate 50 μm and 80 μm in width of microvessel, respectively. (For interpretation of the references to colour in this figure legend, the reader is referred to the Web version of this article.)

Finally, we produced the hepatic lobule-like structure with a continuous and controlled microvascular network. To this end, both “microvessel” channels (=Ø80 μm) and “central vein” channel (=Ø200 μm) were directly patterned in the HepG2 cell-containing hydrogel within a fully assembled microfluidic chip, and then seeded with RFP-HUVECs to achieve a complete vascular network of the hepatic lobule (Fig. 5A). Fluorescence images show that RFP-HUVECs formed radial “microvessels” and a vertical “central vein” in the hydrogel region on day 5 (Fig. 5B). Furthermore, to visualize 3D aggregates of HepG2 cells in the hydrogel region, the cells were stained with a fluorescence dye, BioTracker 488 Green, before cell encapsulation in the hydrogel. 3D images revealed that a vascularized hepatic lobule-like structure was largely constructed on day 5, though some “microvessels” remained missing in the higher layers (Fig. 5C). By contrast, the vascularized hepatic lobule-like structure was completed on day 9, including three layers of radial “microvessels”, vertical “central vein”, and “3D high cell-density hepatic tissue”, though the fluorescence intensity in HepG2 cells decreased over time probably owing to their proliferation. In long-term culture, the constructed microvessels in the hydrogel region partly regressed on day 13 (Fig. S7).

**Fig. 5:**
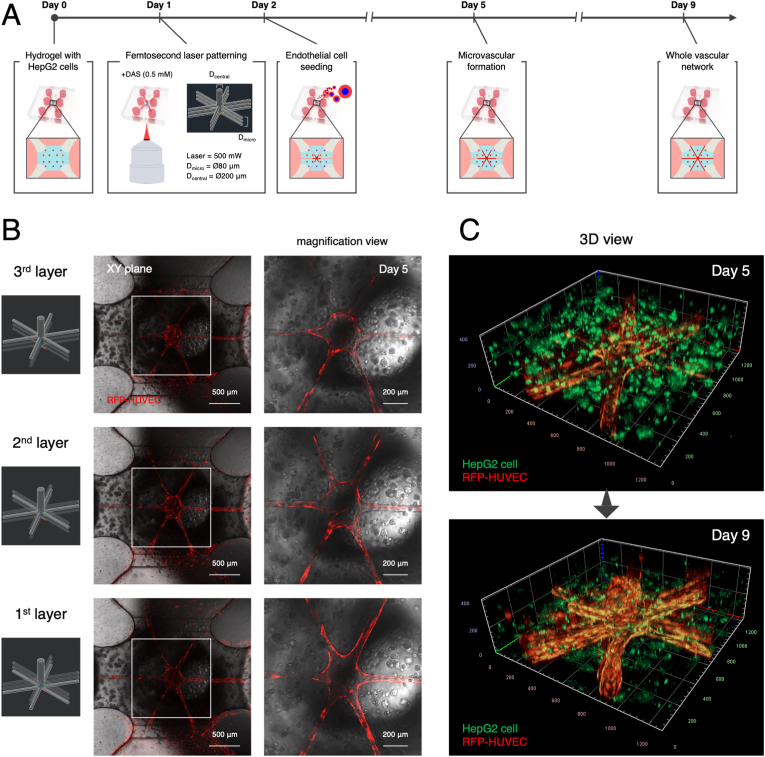
Construction of whole vascular network in the live-on-chip. (A) Experimental timeline for the fabrication of the liver-on-chip. Three layers of “microvessels” and a vertical “central vein” channel were constructed in the hydrogel region. (B) Fluorescence images of RFP-HUVECs (red) in the laser-patterned microchannels in the 1^st^, 2^nd^, and 3^rd^ layers on day 5. Scale bars: 500 μm and 200 μm. (C) 3D images of hepatic lobule-like structure, including HepG2 cells (green) and microvascular network (red), in the hydrogel region on days 5 (upper) and 9 (lower). (For interpretation of the references to colour in this figure legend, the reader is referred to the Web version of this article.)

### 3.4 Functionality assays of liver-on-chip

Finally, in order to assess liver-related functions of our liver-on-chip model, albumin and urea levels in culture medium were measured. In the present study, the following three groups were compared to investigate the effect of the laser-patterned microchannels on liver functions: (i) HepG2 cell-containing hydrogel without microchannels (=Channel(−)), (ii) that with microchannels (=Channel(+)), and (iii) that with microchannels vascularized by HUVEC (=Channel(+HUVEC)) (Fig. 6A). As for albumin production, there was an increase in albumin levels in all groups from day 5–9 probably due to the proliferation of HepG2 cells in the hydrogel (Fig. 6B). Notably, Channel(+) and Channel(+HUVEC) groups exhibited roughly 2.1- and 2.2-fold increases in albumin levels compared to Channel(−) group on day 5 and roughly 1.6- and 1.7-fold increases on day 9, respectively, though these differences were not statistically significant. In addition, urea production was significantly upregulated in Channel(+) and Channel(+HUVEC) groups compared to Channel(−) group on day 9 (Fig. 6C). Specifically, Channel(+) and Channel(+HUVEC) groups show roughly 2.4- and 3.2-fold increases compared to Channel(−) group on day 9. Our results suggest that the presence of laser-patterned microchannels and subsequent vascularization can contribute to the improvements in liver function presumably owing to enhanced delivery of nutrients and oxygen to the entire hydrogel region or paracrine interactions between HepG2 cells and HUVECs.

**Fig. 6:**
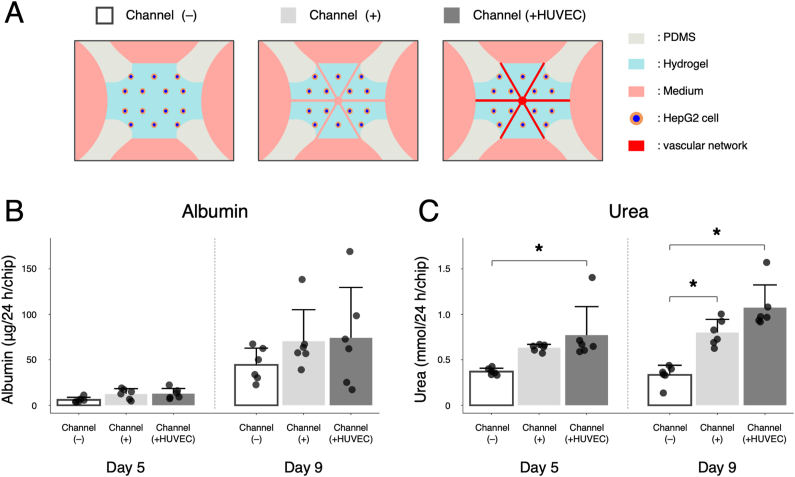
Liver functionality assays of the liver-on-chip. (A) Schematic illustration of three different groups without laser-patterned microchannels (= Channel (−)), with microchannels (= Channel (+)), and with microchannels vascularized by HUVEC (= Channel (+HUVEC)). (B) The level of albumin production (μg/24 h/chip) on days 5 and 9. (C) The level of urea production (mmol/24 h/chip) on days 5 and 9. Data represent the mean ± SD (n = 6/group). ∗*p* < 0.05 (two-way ANOVA with the post hoc Tukey's honestly significant difference test).

To further assess the potential of the liver-on-chip as a drug-induced hepatotoxicity model, acetaminophen (APAP) was administered to the liver-on-chip. APAP overdose is well documented to cause acute liver failure as a major example of drug-induced liver injury [4]. Firstly, a subtoxic concentration of APAP to HepG2 cells was estimated in 2D culture model. 24 h after plating HepG2 cells in a 96-well plate, the cells were treated with APAP for 24 h at different concentrations, ranging from 1 to 80 mM. PrestoBlue assay revealed that a lethal concentration 50 % (LC_50_) of APAP, where the metabolic activity decreased down to 50 % compared to the control group without APAP, was roughly 4 mM (Fig. S8). Based on the result in 2D, APAP at 4 mM was administered to the liver-on-chip model on day 9 where the vascularized hepatic lobule-like structure was constructed. After 24 h of the APAP treatment, cell metabolic assay (i.e., PrestoBlue assay) and live/dead staining were performed on day 10 (Fig. 7A). As expected, PI^+^ damaged cells were detected in the APAP (+) group, whereas only a few PI^+^ damaged cells were observed in the APAP (−) group (Fig. 7B). Moreover, magnified-view images revealed that both HepG2 cells in the cell aggregates and HUVECs in the laser-patterned microchannels appeared to be damaged by the APAP treatment (Fig. 7B, arrowheads). Quantitative analyses also revealed reduction in the live cells (%) based on live/dead staining and the cell metabolic activity (%) based on PrestoBlue assay by the addition of APAP (Fig. 7C and D), suggesting potential drug-induced hepatotoxicity of the vascularized hepatic lobule-like tissue in our model.

**Fig. 7:**
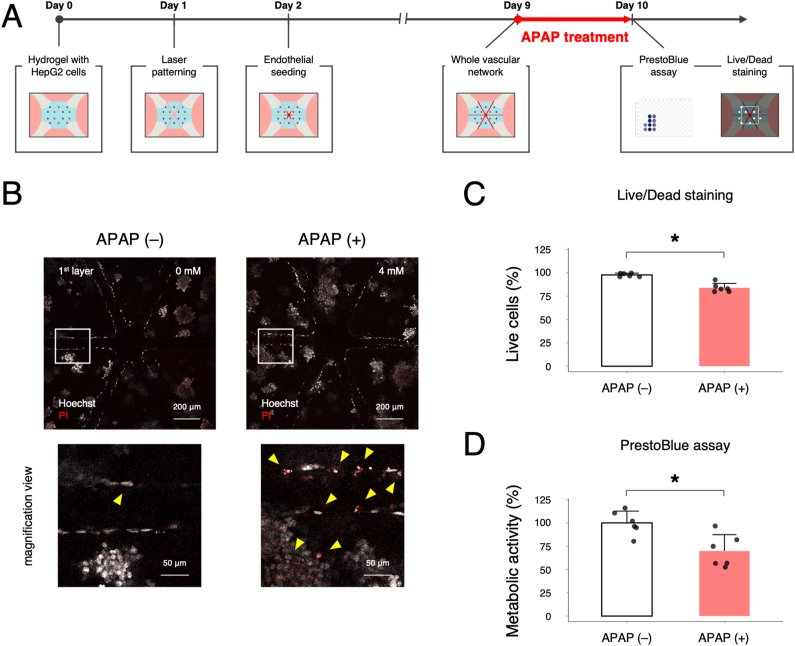
Drug-induced hepatotoxicity test using acetaminophen (APAP). (A) Experimental timeline for the APAP treatment. (B) Fluorescence images of Hoechst (for live cell nuclei, white) and PI (for damaged cell nuclei, red) in the APAP (−) and APAP (+) groups on day 10. Magnification-view images show the white boxes in the above images. Yellow arrowheads indicate PI^+^ damaged cells. Scale bars: 200 μm and 50 μm. (C, D) Quantitative analyses of live cells (%) based on live/dead staining (C) and cell metabolic activity (%) based on PrestoBlue assay (D). Data represent the mean ± SD (n = 6/group). ∗*p* < 0.05 (Student's *t*-test). (For interpretation of the references to colour in this figure legend, the reader is referred to the Web version of this article.)

## 4 Discussion

### 4.1 Realization of millimeter-scale hepatic lobule-like structure with micron-scale resolution

In the present study, we constructed the minimum functional unit of the liver, which is known as the hepatic lobule, whose hexagonal dimensions are considered as Ø1.5–2.5 mm in hexagonal diameter and 0.3–0.9 mm in height in the human liver [36]. To mimic the hepatic lobule *in vitro*, various techniques have been applied over the last decade. Focusing on its millimeter-scale large structure, some studies have employed extrusion-based [19,37] and stereolithography-based [17,18] techniques to develop *in vitro* hepatic lobule models. However, these models failed to produce a continuous microvascular network, ranging from large vessels down to microvessels (<Ø100 μm). On the other hand, focusing on microvessels, other studies have utilized laser-based techniques to construct microvascular structures [[25], [26], [27]]. However, these studies focused only on vasculatures without the use of hepatic cells. To the best of our knowledge, the present study is the first attempt to construct a hepatic lobule-like structure *in vitro* by femtosecond laser patterning. In pursuit of increasing the efficacy of the process within a hydrogel containing hepatic cells, we combined the laser-based technique and a photosensitizer (DAS). Our results demonstrated that the use of DAS improved the construction of millimeter-scale hepatic lobule-like structure, whose size is roughly Ø2.3 mm × 1 mm, without compromising the high resolution of the laser patterning. Interestingly, though DAS was initially developed as a photoinitiator to initiate photo-crosslinking reaction [34], it was found to act as a photosensitizer during laser patterning. In addition, considering that the sizes of conventional hepatic lobule models were Ø1 mm × 0.5 mm by extrusion bioprinting [19] and Ø1 mm × 0.2 mm by stereolithography [17], our proposed strategy offers a better processing technology in terms of dimensions as well as resolution. Taken together, we believe that our proposed strategy and findings will provide valuable guidelines to develop not only liver-on-chip models but also other organ-on-chip models that possess millimeter-scale functional units with micron-scale complexities of the *in vivo* human organs, such as villus in the intestine, glomerulus in the kidney, alveolus in the lung, and neuronal tissue in the brain [3,38].

In addition, there is room for improvement in the hepatic cell density due to light scattering of the laser while patterning within the cell-containing hydrogel. The *in vivo* liver is estimated to contain hepatocytes at roughly 150 × 10^6^ cells/mL [39]. Although the initial hepatic cell density (=0.5 × 10^6^ cells/mL) in our model was lower than that in the *in vivo* liver, HepG2 cells proliferated rapidly in our model over time. For this reason, our approach can be applied to models using proliferative hepatic cells, such as induced pluripotent stem cell (iPSC)-derived hepatic progenitor cells [40]. Moreover, a recent work using a light-based technique demonstrated that the use of specialized compounds can contribute to processing a high cell-density tissue by enabling a 10-fold reduction in light scattering [41]. The use of such compounds will potentially be helpful to increase the initial HepG2 cell density in our model. Besides, we also underline there is a size discrepancy in diameter between the constructed microvessels (=Ø80 μm) in our model and liver sinusoids (=Ø5–10 μm). Considering the fact that capillaries, including the liver sinusoids, are known to represent a high surface area-to-volume ratio, which enables an effective nutrients and waste exchange, the size discrepancy in microvessel diameter may limit a long-term maintenance of the constructed hepatic lobule-like structure in our model. Our future work will focus on the size discrepancy in the diameter to improve the physiological relevance of our model.

### 4.2 Upregulation in liver functions in the presence of HD laser-patterned microchannels

Liver functionality assays revealed that the laser-patterned microchannels improved albumin and urea productions in our liver-on-chip model. In general, the diffusion limit of nutrients and oxygen is considered to be 100–200 μm [42]. Considering that our microfluidic chip has a wide hydrogel region (=2000 μm width), which is much longer than the distance of the diffusion limit, HepG2 cells in the central region of the hydrogel may suffer from lack of adequate nutrients and oxygen without the laser-patterned microchannels, possibly leading to the decreased liver functions. Our result highlights the importance of the microchannels that can deliver nutrients and oxygen to the entire tissue in order to maintain millimeter-scale large tissues *in vitro*.

As expected, our results demonstrated the presence of vascularized laser-patterned microchannels also exhibited higher albumin and urea productions. The improvements in liver function in our model may be partially attributed to interactions between hepatic cells and ECs through various angiocrine signaling pathways [[43], [44], [45], [46]]. Indeed, it has been widely reported that coculture models using hepatic parenchymal cells (i.e., hepatocytes) and non-parenchymal cells (e.g., ECs) improve liver-related functions compared to monoculture of hepatocytes in 2D [47] and 3D models [48,49]. Furthermore, focusing on patterned vascular structure, a recent work utilizing extrusion bioprinting demonstrated that a radially patterned hepatic cell/EC construct in bioink showed higher albumin and urea levels in a long-term culture compared to a random cell-mixture construct [19]. Similarly, another recent work utilizing microtissue molding demonstrated that a liver graft with patterned EC cords in fibrin showed a significantly higher level of human albumin after being implanted in mice than a random cell-mixture liver graft [8]. These findings support the importance of patterned vascularized microchannels in liver models in terms of liver function.

### 4.3 Recreation of acute liver failure in liver-on-chip

Our liver-on-chip model showed potential cellular damage in response to APAP, which is consistent with the obtained results of the *in vivo* human liver [50]. In general, drug-metabolizing enzymes, cytochrome P450s (CYPs) 2E1 and 1A2 in hepatocytes are known to convert APAP into a toxic intermediate N-acetyl-p-benzoquinoneimine (NAPQI) [4,51]. Subsequently, NAPQI reacts with sulfhydryl groups on cysteine and lysine residues, generating adducts with proteins in mitochondria in cells, leading to mitochondrial dysfunction and cell death. In the present study, we observed not only damaged HepG2 cells but also damaged HUVECs after the APAP treatment. Although the detailed mechanisms remain unclear, several *in vivo* studies have shown APAP is toxic not only to hepatocytes but also to ECs, as evidenced by damaged morphology of ECs and increased vascular leakage (e.g., red blood cells) into the space of Disse [52,53]. In one possible scenario, NAPQI converted by HepG2 cells may be released from damaged HepG2 cells, potentially resulting in the HUVEC cytotoxicity in our model. In another scenario, a recent *in vivo* study demonstrated that serum amyloid-A produced by hepatocytes plays a role in promoting liver sinusoidal injury in APAP-induced injury, causing EC damage and platelet aggregation [54]. Accordingly, the reduction in cell viability by the APAP treatment in our study suggests that our model may retain liver-specific CYP activities and potentially have the ability to recreate the *in vivo* scenario of the acute liver failure induced by drug. Taken together, our result suggests that our liver-on-chip model has potential as a tool for preclinical pharmaceutical and toxicological studies. While this work focused on the technological proof of concept and used simple PDMS-based chips, PDMS is known to adsorb and absorb a wide range of chemical drugs, which may influence experimental results, especially for drug testing applications. In the future, switching to different materials with low adsorptive capacity, such as polystyrene, poly(methyl methacrylate), polycarbonate, and cyclic olefin copolymer, would be important [55].

### 4.4 Limitations and future perspectives

In the present study, we employed HepG2 cell and HUVEC to construct a vascularized hepatic lobule-like structure because of their availability and simplicity of use. However, we underline that HepG2 cells and HUVECs do not fully represent biological features of human hepatocyte and liver sinusoidal EC (LSEC). Wilkening et al. reported that gene expression of some CYP isoforms in HepG2 cell was lower than that in primary human hepatocyte (PHH) [56]. We believe that PHH is one of the most ideal cell sources to achieve a physiologically relevant model for drug testing and disease modeling. On the other hand, LSEC exhibits fenestration on their surface, which allows an intensive interaction between sinusoidal blood and parenchymal cells, facilitating oxygenation of hepatocytes and enhancing hepatocytes exposure to macromolecules from the portal circulation [57,58]. Nevertheless, HUVEC lacks this typical fenestration on their surface. For these reasons, future work will be needed to improve our model by the use of PHH and LSEC to further mimic the physiological microenvironment of the liver.

As for disease modeling, it will be interesting to incorporate other non-parenchymal cell types of the liver, other than EC. It is widely known that one of the major liver diseases, liver fibrosis is induced by interactions between hepatocytes and non-parenchymal cells, such as hepatic stellate cells (HSC), Kupffer cell, and biliary epithelial cells [59]. The hallmark of liver fibrosis is excess accumulation of extracellular matrix (e.g., type I collagen), eventually leading to organ dysfunction. In this scenario, HSC is considered as the major fibrogenic cell in progression of liver fibrosis. Once liver is injured, HSCs transdifferentiate into a fibrogenic myofibroblasts, resulting in production and accumulation of excess fibrous extracellular matrix [60]. In addition, Kupffer cells act as the major source of inflammatory mediators, such as tumor necrosis factor-α [61], which is known as a mediator of liver diseases in terms of hepatic cell death, inflammation, and proliferation [62]. Biliary epithelial cells are also an interesting cell source to recreate pathological cholestasis scenario, which is emerging as a leading cause of liver injury and fibrosis [63]. Furthermore, iPSC-derived hepatic cells are also attractive cell sources in future study [40,64,65]. We believe that the use of these iPSC-derived cells will offer a promising tool to identify an appropriate drug for each individual patient. Since there is no reliable biomarker for the liver toxicity at the moment, prediction and avoidance of unpredictable drug-induced liver failure is one of the most important objectives of using *in vitro* liver models for personalized medicine [66,67]. Collectively, future work will focus on introducing these non-parenchymal cell types to our liver-on-chip in order to further improve the complexity of the model toward pathologically relevant microenvironments.

In addition to the above cell heterogeneity, a spatial functional heterogeneity in the hepatic lobule, known as liver zonation, is one of the most important features of the liver [51,68]. The liver zonation largely relies on blood circulation from the periportal zone to the pericentral zone in the hepatic lobule, which creates gradients of oxygen, nutrients, and hormones, leading to the spatial functional heterogeneity. Our future research will go in the direction of incorporating physiological flow conditions in our model to realize the liver zonation. Moreover, the flow conditions could be helpful to achieve a long-lasting hepatic lobule structure *in vitro* in terms of nutrients and waste exchange.

Another attractive aspect of our liver-on-chip is the possibility to use the vascularized hepatic lobule-like unit as a building block. Since we have successfully constructed the functional unit of the liver with a continuous microvascular network in the present study, we believe it may be feasible to construct a clinically effective liver graft by assembling the hepatic lobule-like structures isolated from microfluidic chips, leading from millimeter-scale tissue to centimeter-scale graft. Taken together, our liver-on-chip model, laser-based strategy, and findings will be helpful for the development of physiological and pathological liver models and possibly for the construction of clinically effective liver grafts in tissue engineering and regenerative medicine.

## 5 Conclusions

We have successfully constructed a hepatic lobule-like structure with a continuous microvascular network via high-definition (HD) laser patterning in the microfluidic chip. In particular, we demonstrate that the use of photosensitizers allows to substantially improve the efficiency of femtosecond laser patterning and greatly contributes to the construction of millimeter-scale biomimetic tissue with micron-scale resolution, such as microvessels, in the liver-on-chip. Importantly, the presented approach allows to produce channels directly within a completely assembled microfluidic chip produced by conventional means. This way it is possible to combine standard off-the shelf devices with a versatile material platform and a flexible high-resolution technology, allowing superior reproducibility and easy adaptation of the 3D design. We believe that our proposed approach can be applied to the development of other organ-on-chip models that possess organ-specific large functional units with microvascular networks. Moreover, another important feature is the construction of a continuous microvascular network, from the microvessels to the central vein, which is a critical route for drug delivery in the liver. Therefore, our liver-on-chip model will be a promising platform for preclinical pharmaceutical and toxicological studies to investigate how drugs are delivered from the blood circulatory system to hepatocytes and how they are metabolized in the liver. Furthermore, our liver-on-chip exhibited major liver functions (i.e., albumin and urea production) and drug-induced hepatotoxicity, which are key evidence that we have realized a functional liver tissue *in vitro*. Collectively, our proposed approach and liver-on-chip will provide highly valuable guidelines for the development of advanced physiological and pathological organ-on-chip models that enable not only to better predict the efficacy and safety of drugs but also to develop new therapeutic strategies for organ diseases.

## CRediT authorship contribution statement

**Masafumi Watanabe:** Writing – original draft, Methodology, Investigation, Funding acquisition, Formal analysis, Data curation, Conceptualization. **Alice Salvadori:** Writing – review & editing, Methodology, Investigation, Funding acquisition. **Marica Markovic:** Writing – review & editing, Methodology, Investigation. **Ryo Sudo:** Writing – review & editing, Methodology. **Aleksandr Ovsianikov:** Writing – review & editing, Supervision, Resources, Project administration, Methodology, Conceptualization.

## Declaration of competing interest

The authors declare the following financial interests/personal relationships which may be considered as potential competing interests:A.O. is also a Co-Founder and CSO of UpNano GmbH. If there are other authors, they declare that they have no known competing financial interests or personal relationships that could have appeared to influence the work reported in this paper.

## Figures and Tables

**figs1:**
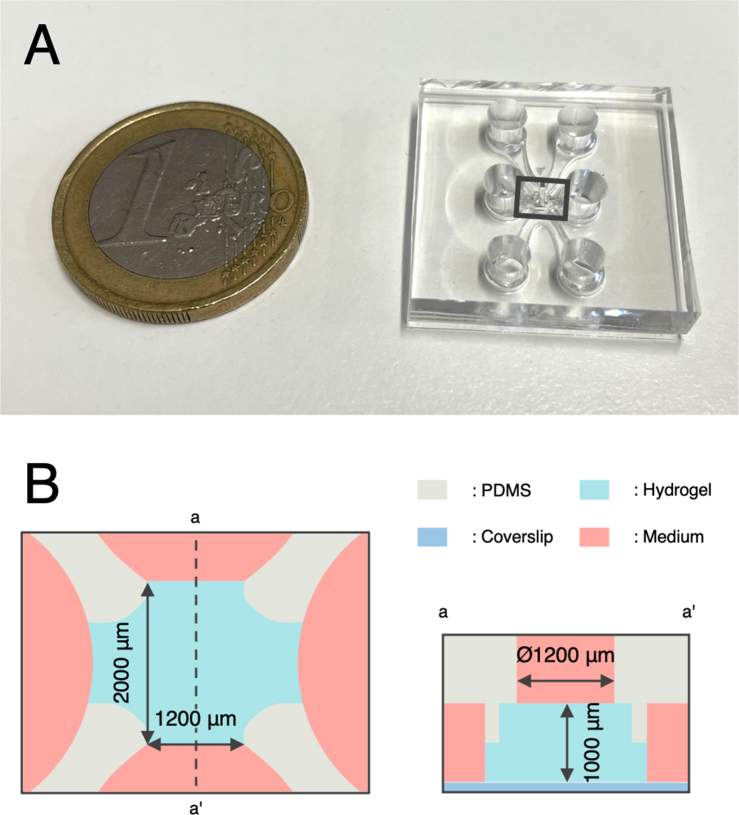
Photograph of microfluidic chip. (A) Photograph of the microfluidic chip (right) and the 1 euro coin (left). (B) Detailed dimensions of the microfluidic chip. The cross-view image corresponds to the dotted line (a—a') in the left image.

**figs2:**
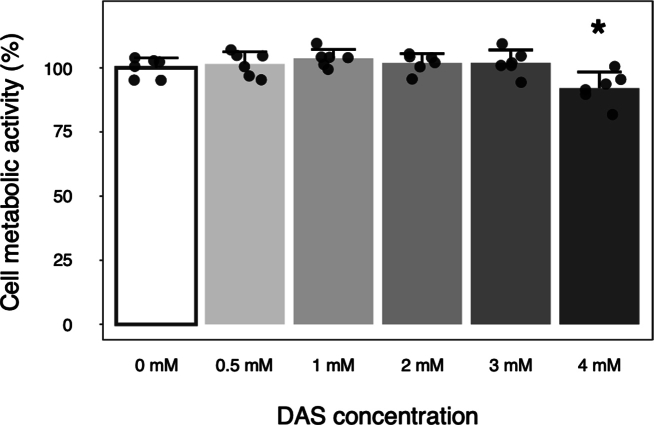
Cell metabolic activity of HepG2 cells after 24 h of incubation with a photosensitizer DAS. PrestoBlue assay was performed to evaluate the metabolic activities of HepG2 cells with different concentrations of DAS (= 0, 0.5, 1, 2, 3, and 4 mM). Data represent the mean ± SD (n = 6/group). ∗*p* < 0.05 (one-way ANOVA with the post hoc Dunnet's test).

**figs3:**
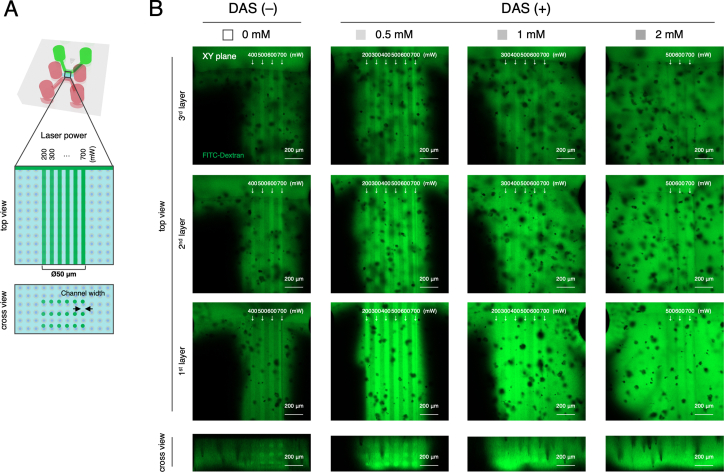
Optimization of DAS concentration for microchannel formation. (A) Schematic illustrations of microchannel formation in the cell-containing hydrogel with different laser powers ranging 200 to 700 mW. (B) Fluorescence images of microchannel formation with different concentrations of DAS at 0, 0.5, 1, and 2 mM. Laser-patterned microchannels were visualized by FITC-Dextran perfusion (2000 kDa, green). Scale bars: 200 μm.

**figs4:**
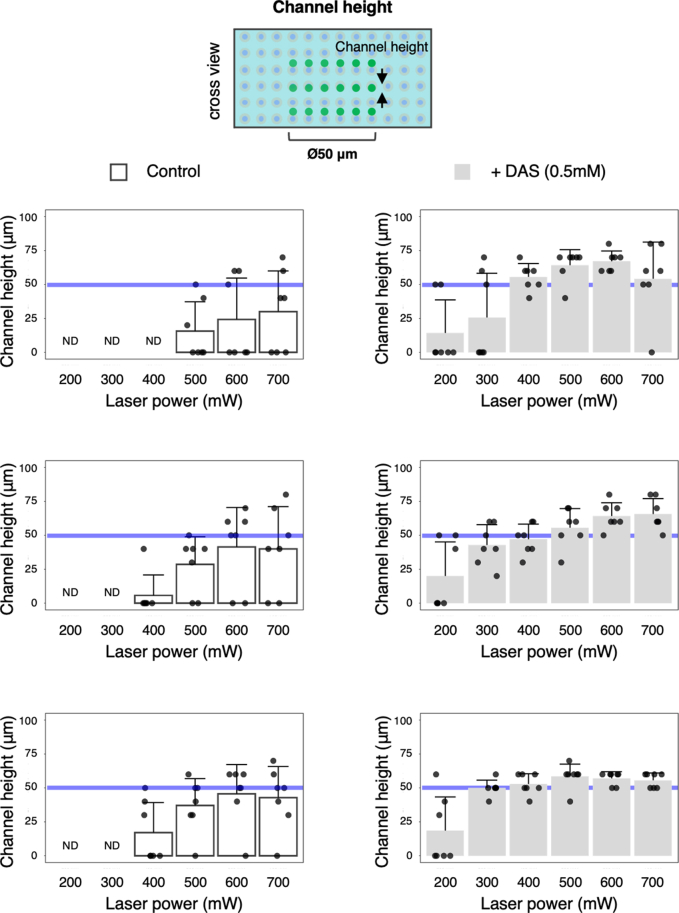
Quantitative analysis of channel height in the cell-containing hydrogel with different laser powers ranging from 200 to 700 mW in the control group without DAS and in the group with DAS at 0.5 mM. Data represent the mean ± SD (n = 7/group). ND indicates "not detected". Blue lines indicate 50 μm in channel height.

**figs5:**
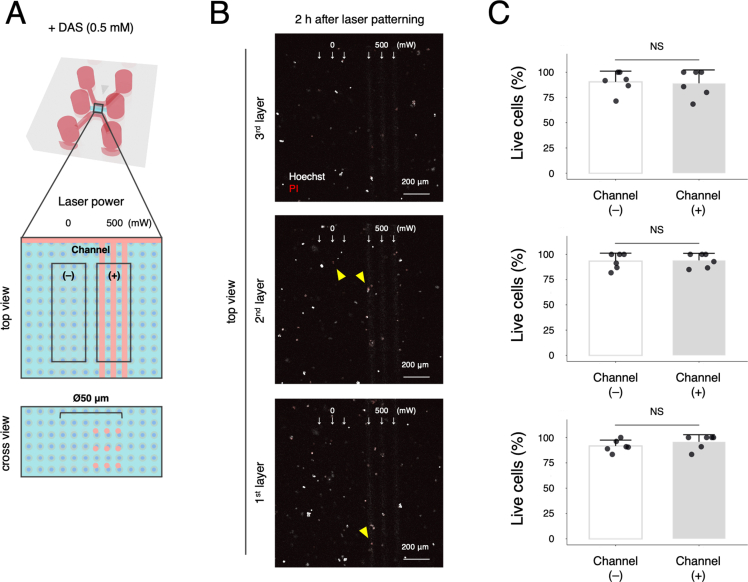
Live/dead staining of HepG2 cells in the hydrogel region after femtosecond laser patterning with DAS. (A) Schematic illustrations of microchannel formation in the cell-containing hydrogel with 500 mW laser power. (B) Fluorescence images of HepG2 cells stained with Hoechst (for live cell nuclei, white) and PI (for damaged cell nuclei, red) in the 1^st^, 2^nd^, and 3^rd^ layers in the hydrogel region 2 h after the laser patterning. Yellow arrowheads indicate PI^+^ damaged cells. Scale bars: 200 μm. (C) Quantitative analysis of live cells (%) based on live/dead staining. Data represent the mean ± SD (n = 6/group). NS indicates "not significant" (Student' *t*-test).

**figs6:**
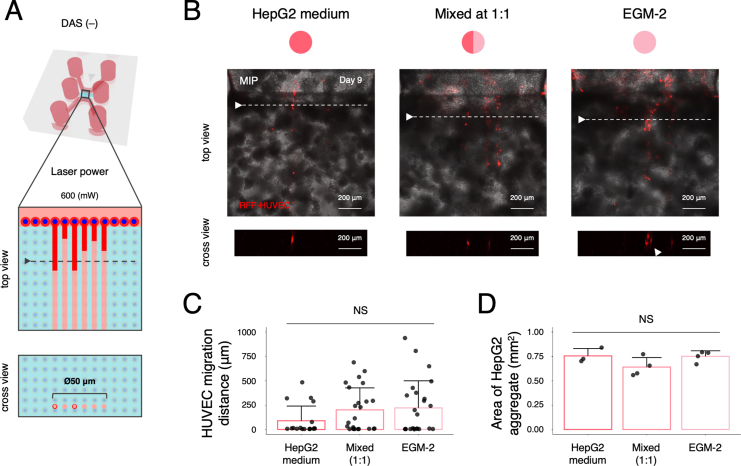
Optimization of culture medium for HepG2 cell and RFP-HUVEC. (A) Schematic illustrations of microvessel formation in the cell-containing hydrogel. RFP-HUVECs were seeded to one side channel of the microfluidic chip. (B) Max intensity projection (MIP) images of RFP-HUVECs in the laser-patterned microchannels under different conditions of culture medium, such as HepG2 medium, EGM-2, and the mixed medium of them at 1:1 ratio on day 9. The cross-view images correspond to the dotted lines in the MIP images. An arrowhead indicates a luminal structure of microvessel. Scale bars: 200 μm. (C) Quantitative analysis of HUVEC migration distance. Data represent the mean ± SD (n > 18/group). ∗*p* < 0.05 (one-way ANOVA with the post hoc Tukey's honestly significant difference test). (D) Quantitative analysis of area of HepG2 cell aggregate. Data represent the mean ± SD (n > 3/group). ∗*p* < 0.05 (one-way ANOVA with the post hoc Tukey's honestly significant difference test). NS indicates "not significant".

**figs7:**
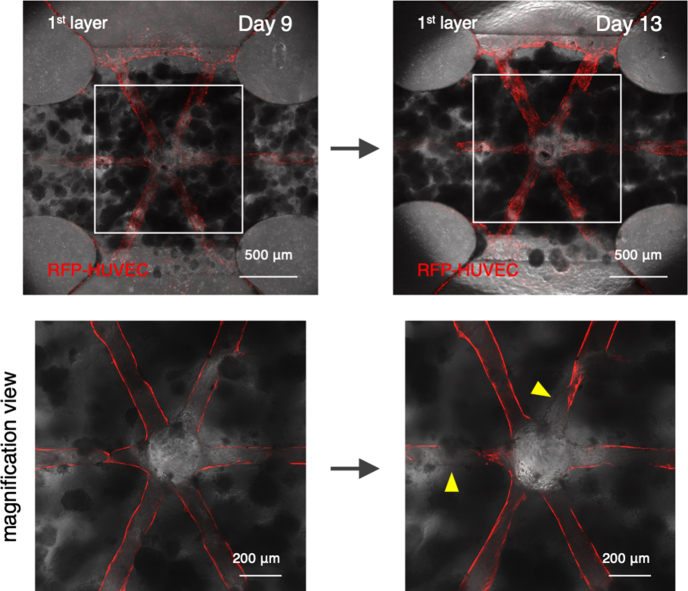
Long-term stability of vascularized hepatic lobule-like structure. Fluorescence images of RFP-HUVECs in the 1^st^ layer in the hydrogel on days 9 and 13. Magnification-view images show the white boxes in the above images. Yellow arrowheads indicate regressed microvessels.

**figs8:**
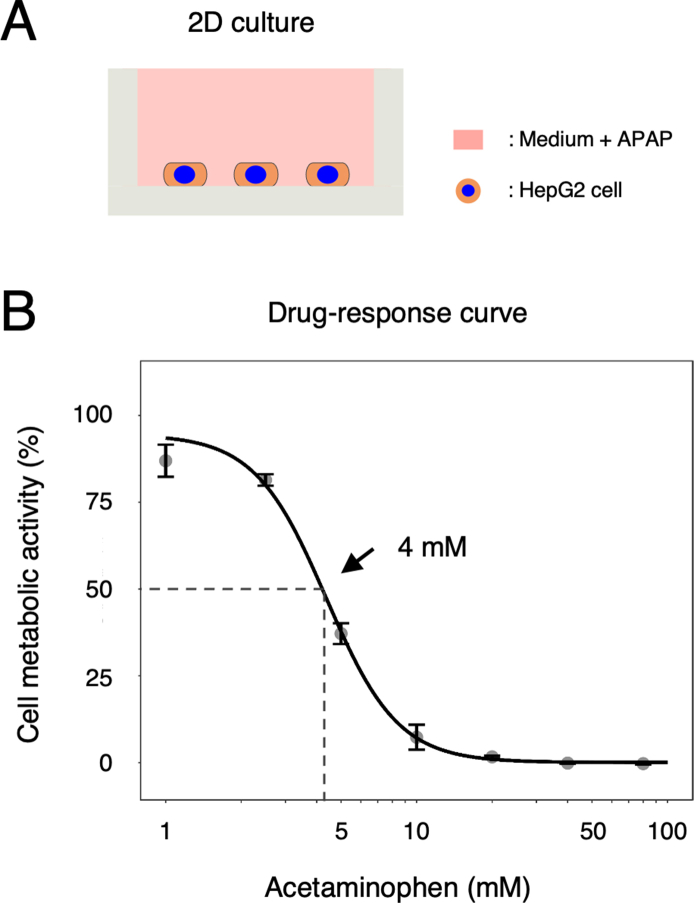
Dose-response curve of acetaminophen (APAP) to HepG2 cell in 2D. (A) Schematic illustration of HepG2 cell culture in 2D. (B) Dose-response curve of APAP. PrestoBlue assay was performed to evaluate the metabolic activity of HepG2 cells with different concentrations of APAP (= 1.0, 2.5, 5.0, 10.0, 20.0, 40.0, and 80.0 mM). Data represent the mean ± SD (n = 4/concentration). The concentration of APAP was estimated to be roughly 4 mM where the cell metabolic activity decreased to 50% (an arrowhead in the graph).

## Data Availability

Data will be made available on request.
