# Supplementary material for: Advanced liver-on-chip model mimicking hepatic lobule with continuous microvascular network via high-definition laser patterning
Source: Mater Today Bio. 2025 Mar 7;32:101643. doi: 10.1016/j.mtbio.2025.101643 (PMC11979415; doi:10.1016/j.mtbio.2025.101643)
Supplement: Multimedia component 1 [file mmc1.docx]

**Supplementary data**

**Advanced liver-on-chip model mimicking hepatic lobule with continuous microvascular network via high-definition laser patterning**

Masafumi Watanabe^a,b,c^, Alice Salvadori^a,b^, Marica Markovic^a,b^, Ryo Sudo^d^,

and Aleksandr Ovsianikov^a,b,*^

^a^ Research Group 3D Printing and Biofabrication, Institute of Materials Science and Technology, Technische Universität Wien (TU Wien), 1060 Vienna, Austria.

^b^ Austrian Cluster for Tissue Regeneration (<https://www.tissue-regeneration.at>).

^c^ Japan Society for the Promotion of Science (JSPS) Overseas Research Fellow.

^d^ Department of System Design Engineering, Keio University, 223-8522 Yokohama, Japan.

***** Corresponding author: Aleksandr Ovsianikov (E-mail: [aleksandr.ovsianikov@tuwien.ac.at](mailto:aleksandr.ovsianikov@tuwien.ac.at))

**Fig. S1.** Photograph of microfluidic chip. (A) Photograph of the microfluidic chip (right) and the 1 euro coin (left). (B) Detailed dimensions of the microfluidic chip. The cross-view image corresponds to the dotted line (a–a’) in the left image.

**Fig. S2.** Cell metabolic activity of HepG2 cells after 24 h of incubation with a photosensitizer, DAS. PrestoBlue assay was performed to evaluate the metabolic activities of HepG2 cells with different concentrations of DAS (= 0, 0.5, 1, 2, 3, and 4 mM). Data represent the mean ± SD (n = 6/group). ∗*p* < 0.05 (one-way ANOVA with the post hoc Dunnett’s test).

**Fig. S3.** Optimization of DAS concentration for microchannel formation. (A) Schematic illustrations of microchannel formation in the cell-containing hydrogel with different laser powers ranging from 200 to 700 mW. (B) Fluorescence images of microchannel formation with different concentrations of DAS at 0, 0.5, 1, and 2 mM. Laser-patterned microchannels were visualized by FITC-Dextran perfusion (2000 kDa, green). Scale bars: 200 μm.

**Fig. S4.** Quantitative analysis of channel height in the cell-containing hydrogel with different laser powers ranging from 200 to 700 mW in the control group without DAS and in the group with DAS at 0.5 mM. Data represent the mean ± SD (n = 7/group). ND indicates “not detected”. Blue lines indicate 50 μm in channel height.

**Fig. S5.** Live/dead staining of HepG2 cells in the hydrogel region after femtosecond laser patterning with DAS. (A) Schematic illustrations of microchannel formation in the cell-containing hydrogel with 500 mW laser power. (B) Fluorescence images of HepG2 cells stained with Hoechst (for live cell nuclei, white) and PI (for damaged cell nuclei, red) in the 1^st^, 2^nd^, and 3^rd^ layers in the hydrogel region 2h after the laser patterning. Yellow arrowheads indicate PI^+^ damaged cells. Scale bars: 200 μm. (C) Quantitative analyses of live cells (%) based on live/dead staining. Data represent the mean ± SD (n = 6/group). NS indicates “not significant” (Student’s *t*-test).

**Fig. S6.** Optimization of culture medium for HepG2 cell and RFP-HUVEC. (A) Schematic illustrations of microvessel formation in the cell-containing hydrogel. RFP-HUVECs were seeded to one side channel of the microfluidic chip. (B) Maximum intensity projection (MIP) images of RFP-HUVECs in the laser-patterned microchannels under different conditions of culture medium, such as HepG2 medium, EGM-2, and the mixed medium of them at 1:1 ratio on day 9. The cross-view images correspond to the dotted lines in the MIP images. An arrowhead indicates a luminal structure of microvessel. Scale bars: 200 μm. (C) Quantitative analysis of HUVEC migration distance. Data represent the mean ± SD (n ≥ 18/group). ∗*p* < 0.05 (one-way ANOVA with the post hoc Tukey’s honestly significant difference test). (D) Quantitative analysis of area of HepG2 cell aggregate. Data represent the mean ± SD (n ≥ 3/group). ∗*p* < 0.05 (one-way ANOVA with the post hoc Tukey’s honestly significant difference test). NS indicates “not significant”.

**Fig. S7.** Long-term stability of vascularized hepatic-lobule like structure. Fluorescence images of RFP-HUVECs in the 1^st^ layer in the hydrogel on days 9 and 13. Magnification-view images in the 1^st^ layer show the white boxes in the above images. Yellow arrowheads indicate regressed microvessels.

**Fig. S8.** Dose-response curve of acetaminophen (APAP) to HepG2 cell in 2D. (A) Schematic illustration of HepG2 cell culture in 2D. (B) Dose-response curve of APAP. PrestoBlue assay was performed to evaluate the metabolic activity of HepG2 cells with different concentrations of APAP (= 1.0, 2.5, 5.0, 10.0, 20.0, 40.0, and 80.0 mM). Data represent the mean ± SD (n = 4/concentration). The concentration APAP was estimated to roughly 4 mM when the cell metabolic activity decreased down to 50 % (an arrow in the graph).
